# A Framework for Protecting and Promoting Employee Mental Health through Supervisor Supportive Behaviors

**DOI:** 10.1007/s41542-023-00171-x

**Published:** 2024-01-30

**Authors:** Leslie B. Hammer, Jennifer Dimoff, Cynthia D. Mohr, Shalene J. Allen

**Affiliations:** 1 Oregon Institute of Occupational Health Sciences, Oregon Health & Science University, Portland, OR, USA; 2 Telfer School of Management, University of Ottawa, Ottawa, Canada; 3 Department of Psychology, Portland State University, Portland, OR, USA

**Keywords:** Workplace mental health, Supervisor support interventions, Psychosocial risk factors

## Abstract

The attention to workplace mental health is timely given extreme levels of burnout, anxiety, depression and trauma experienced by workers due to serious extraorganizational stressors – the COVID-19 pandemic, threats to climate change, and extreme social and political unrest. Workplace-based risk factors, such as high stress and low support, are contributing factors to poor mental health and suicidality (Choi, 2018; Milner et al., 2013, 2018), just as low levels of social connectedness and belonging are established risk factors for poor mental health (Joiner et al., 2009), suggesting that social support at work (e.g., from supervisors) may be a key approach to protecting and promoting employee mental health. Social connections provide numerous benefits for health outcomes and are as, or more, important to mortality as other well-known health behaviors such as smoking and alcohol consumption (Holt-Lundstad et al., 2015), and can serve as a resource or buffer against the deleterious effects of stress or strain on psychological health (Cohen & Wills, 1985). This manuscript provides an evidence-based framework for understanding how supervisor supportive behaviors can serve to protect employees against psychosocial workplace risk factors and promote social connection and belongingness protective factors related to employee mental health. We identify six theoretically-based Mental Health Supportive Supervisor Behaviors (MHSSB; i.e., emotional support, practical support, role modeling, reducing stigma, warning sign recognition, warning sign response) that can be enacted and used by supervisors and managers to protect and promote the mental health of employees. A brief overview of mental health, mental disorders, and workplace mental health is provided. This is followed by the theoretical grounding and introduction of MHSSB. Suggestions for future research and practice follow, all with the focus of developing a better understanding of the role of supervisors in protecting and promoting employee mental health in the workplace.

## Statement of Relevance to Special Issue

Mental health of employees has never before received greater attention from researchers, employers, and popular media in the United States (U.S.). Spurred by the negative impact of the coronavirus (COVID-19) pandemic on mental health (Dozois, 2021), many employers are placing greater emphasis on inclusive health benefits, policies, and programs that are focused on promoting the mental health and well-being of employees (e.g., Greenwood & Anas, 2021). Mental disorders (i.e., conditions that affect one’s thinking, feeling, mood, and behavior; American Psychiatric Association, 2022) are experienced by over 20% of the population (SAMHSA, 2021), and the World Health Organization (WHO) has identified depression as the leading cause of disability around the world (WHO, 2021; 2022). Workplace-based risk factors, such as high stress and low support, are contributing factors to poor mental health and suicidality (Choi, 2018; Milner et al., 2013, 2018), and may put employees at a greater risk for mental disorders (American Psychiatric Association, 2022).

Given the amount of time people spend working, it is important to understand workplace mental health risk factors, as well as the role of the workplace in promoting and protecting mental health. There is a need for evidence-based workplace mental health interventions, and this paper offers a framework for the development of supervisor behavioral strategies, referred to as Mental Health Supportive Supervisor Behaviors (MHSSB), that offer promise for future research and practice aimed at improving population mental health. Thus, this paper reviews the role of the supervisor in supporting the mental health of employees, and provides a framework for understanding workplace-supportive strategies as approaches that prevent risk factors and promote protective factors related to employee mental health. We identify theoretically-based MHSSB that can be enacted and used by supervisors and managers to protect and promote the mental health of employees. A brief overview of mental health, mental disorders, and workplace mental health is provided. This is followed by the role of the supervisor in promoting social connections at work and the theoretical grounding and introduction of MHSSB. Suggestions for future research and practice follow, all with the focus of developing a better understanding of the role of supervisors in protecting and promoting employee mental health in the workplace.

## Mental Health and Mental Disorders

The World Health Organization (WHO) defines mental health as a “state of well-being in which the individual realizes [their] own abilities, can cope with the normal stresses of life, can work productively and fruitfully, and is able to contribute to [their] community (WHO, 2016, see specifically the chapter titled “Target 3.4: Suicide”). Mental health is considered integral not only to overall health and well-being (i.e., happiness, life satisfaction, and positivity; Diener, 1984), but also to an individual’s ability to be productive and perform at work, form strong social connections with others, and contribute to society more broadly (WHO, 2016). Mental health is experienced on a continuum (see Fikretoglu et al., 2017; Kelloway et al., 2023; Keyes, 2002), typically ranging from “mental health” (see above definition from the WHO) to “mental illness”, which refers to a diagnosable psychological disorder characterized by dysregulation of mood, thought, and/or behavior (American Psychiatric Association, 2013). Note that the terms “mental illness” and “mental disorder” are often used interchangeably and have slightly nuanced meanings globally and across clinical settings (see the Diagnostic and Statistical Manual of Mental Disorders [DSM-5-TR] from the American Psychiatric Association, 2022; see the International Classification of Diseases 11th Revision [ICD-11] from the WHO, 2021). Here we use the term ‘mental disorder’ to remain consistent with the terminology used by the WHO (2021).

Given that mental health status exists on a continuum, when mental health is compromised, people may experience states of poor mental health that are not necessarily clinically diagnosable, such as psychological strain (i.e., a state of distress and tension; Lazarus & Folkman, 1984) or burnout (i.e., a state of emotional, mental, and sometimes physical exhaustion due to prolonged work-related stress; Maslach & Jackson, 1981), that affect how they feel, think, and behave (WHO, 2004; 2018). If not ameliorated for a prolonged period of time, there is a possibility that poor mental health states (e.g., chronic psychological strain) may put someone at an increased risk of developing a diagnosable mental disorder such as generalized anxiety disorder or depression (see the DSM-5-TR from the APA; 2022; see the ICD-11 from the WHO, 2021). Although a complex combination of genetic and biological factors put people at a heightened risk for experiencing states of poor mental health or developing a mental disorder (Mayo Clinic, 2022), environmental and social factors (e.g., financial security and social support) also play a critical role. Environmental conditions associated with work may be particularly relevant to protecting and maintaining mental health (Mayo Clinic, 2022), or serving as risk factors for poor mental health (e.g., job strain; Niedhammer et al., 2021).

## Mental Health: Prevalence, Stigma, and Seeking Care

Recent data estimate that as many as 1 in 4 people experience a mental disorder each year (NIMH, 2022; WHO, 2022). Alarmingly, despite the high prevalence of mental disorders, less than 50% of people will seek out or receive formal support and treatment (National Alliance on Mental Illness, 2022). The concomitant challenges of not seeking treatment for mental disorders can be attributed to factors outside a person’s control; for example, out of network providers, financial costs to follow-through with receiving care, and the societal stigma (i.e., a negative social attitude associated with a characteristic of an individual; American Psychological Association, 2023a; Stone et al., 2023) of pursuing help and support. This is particularly concerning given that mental disorders almost always require professional intervention and ongoing treatment to successfully manage or eliminate symptoms (National Alliance on Mental Illness, 2022).

With the hesitancy of people to seek and receive treatment, many employees experience the negative consequences of poor mental health (for a review see Dimoff & Kelloway, 2019a). Although mental health disorders are protected under the Americans with Disabilities Act (ADA), protection is often only afforded to those who disclose their diagnosis – something that only approximately half of American workers say they feel comfortable doing (American Psychiatric Association, 2019). As many as 1 in 3 workers are worried about work-related consequences if they seek mental health care (American Psychiatric Association, 2019). Employees are often unable to, or afraid of, disclosing their mental health-related diagnosis to their employer or, more often, Human Resources personnel or departments, and thus, may not benefit from accommodations and formal support from their workplace to which they are legally entitled. Understanding how the workplace can create an environment to protect and promote employee mental health is critical for addressing public policy, scholarly research, and workplace practices that positively contribute to population mental health.

## Mental Health and the Role of Work

Recent calls by organizational scholars have identified the role of the workplace in affecting, and being affected by, worker mental health (e.g., Gilmer et al., 2023; Hammer et al., 2022; Kelloway et al., 2023; Rosado-Solomon et al., 2023)—both positively and negatively. Work can serve as a source of physical and psychological risk through unsafe working conditions, interpersonal conflicts, and excessive work hours (e.g., Niedhammer et al., 2021; Sawhney et al., 2023; Schnall et al., 1994). Work can also serve as a source of mental health promotion and protection (Hammer et al., 2022; Rugulies et al., 2023).

When work conditions are unfavorable and psychosocial hazards/risk factors such as high demands, low control, and low support exist, work can cause, facilitate, and exacerbate poor mental and physical health such as psychological strain, burnout, and depression (e.g., Niedhammer et al., 2021). The job strain model (i.e., high demands and low control), and low social support have long been identified as consistent predictors of poor physical and psychological health (Karasek, 1979; Niedhammer et al., 2021; Rugulies et al., 2023). The role of poor social support, in particular, was integrated into the original job strain model (Johnson & Hall, 1988), which was later expanded to include new forms of control, additional aspects of work intensification, and expanded social relations (Lovejoy et al., 2021). In a meta-analysis of 72 reviews between the years of 2000–2020, Niedhammer et al. (2021) further confirmed the significant relationships between job strain and social support with cardiovascular disease and mental health challenges, including depression.

Thus, a number of workplace psychosocial hazards (e.g., low social support, low autonomy, low recognition, high job demands, excessive workload, mistreatment, illegitimate tasks, role conflict, role ambiguity) have been related to mental health outcomes (e.g., Choi, 2018; Demerouti et al., 2001; Milner et al., 2013, 2018; Niedhammer et al., 2021; Yarker et al., 2022), including elevated risk of death by suicide and suicidal behaviors (e.g., Greiner & Arensman, 2022; Milner et al., 2018), which is quickly becoming one of the leading causes of deaths among young adults (SAMHSA, 2021). We argue that evidence-based workplace redesign interventions are needed to target these psychosocial risk factors by increasing worker schedule control and voice, moderating job demands, and by training supervisors and managers on increasing social support at work (Hammer et al., 2023a; Lovejoy et al., 2021). While evidence exists for the associations over time between these psychosocial risk factors and psychological and physical health (Niedhammer et al., 2021), the systematic evaluation of interventions incorporating such organizational-approaches is limited, especially in terms of impacts on mental health outcomes. Some promise exists, however, for effectively improving psychological well-being through supervisor support training (e.g., Hammer et al., 2011; Hammer et al., 2021; Kossek et al., 2019).

Furthermore, despite decades of research on workplace hazards relating to psychological health and well-being of workers, organizational scholars argue that there is a dearth of research specifically focused on evidence-based workplace mental health interventions that take organizational-level approaches, as opposed to individual-approaches (Anger et al., 2023; Aust et al., 2023; Rosado-Solomon et al., 2023). Yet, the recent American Psychological Association. Work in America Study found that 92% of employees reported that it was very or somewhat important to them to work for an organization that provides support for mental health (American Psychological Association, 2023b).

Work provides people with the opportunity to fulfill basic social and economic needs (see Deci & Ryan, 1985) and thus is an ideal point of intervention. Given that work can serve as a protective factor against poor mental health (e.g., psychological strain; burnout) and certain mental disorders (e.g., depression), a promising avenue for workplace interventions that impact employee mental health is through enhancing social connections at work. In fact, the recent U.S. Surgeon General (2023) Advisory on the Healing Effects of Social Connection and Community identifies the workplace as a resource providing a sense of belonging and inclusion that can reduce the detrimental effects of low levels of social connectedness, including poor mental health, making it a valuable target.

Only recently have organizational scholars and practitioners begun to understand and develop interventions that address workplace conditions/organizational factors that contribute to decrements in mental health of workers (e.g., Dimoff & Kelloway, 2016; Thomson et al., 2023). Thus, we argue that reducing psychosocial hazards in the workplace is critical, and one way to do this is through positive resources that promote and protect employees’ mental health, such as increasing supervisor social support. The workplace is a “missing link” in protecting and promoting the mental health of our population, and is an important point of intervention that has been overlooked in the past (American Psychological Association, 2023b; Hammer et al., 2022).

## Belongingness and Loneliness at Work: The Case for Increasing Supervisor Support

Loneliness and social disconnection at work predict lower job performance (Lam & Lau, 2012; Ozcelik & Barsade, 2018), and reduced organizational citizenship behaviors (Lam & Lau, 2012), as well as reduced organizational commitment (Ayazlar & Güzel, 2014; Bartholomeusz et al., 2021). Furthermore, loneliness is related to increased absenteeism and turnover intentions at work (Bowers et al., 2022). Workplace loneliness also explained more variance in mental health outcomes relative to incivility when controlling for perceived work stress in a recent study by Gilmer et al. (2023), suggesting the importance of meaningful social relationships at work, such as through supervisor social support. This research is consistent with findings that manager communication lessened the negative effects of isolation, due to hybrid work during COVID, on mental health symptoms (Sawhney et al., 2023). Perceived social connection and belongingness at work are also related to lower levels of post-traumatic stress symptoms (Britt et al., 2021).

People have a fundamental need to belong which is critical to our survival (Baumeister & Leary, 1995), and this need is satisfied in two ways: by frequent contact or interaction with others; and by perceiving an interpersonal bond or relationship that is stable, caring, and enduring (Baumeister & Leary, 1995). When these needs are unmet, people experience loneliness, which is thought to be a painful signal of relationship deficits, with potentially dire consequences for mental and physical health (Perlman & Peplau, 1981). In addition to the noted workplace outcomes associated with loneliness, compelling evidence for the costs of loneliness was presented in meta-analytic conclusions documenting that loneliness not only predicts early onset mortality and disease susceptibility, but is comparable to previously-established risk factors such as smoking, cardiovascular disease, and obesity (Holt-Lunstad et al., 2015; Holt-Lunstad, 2021). It is also an identified risk factor for suicidality (Joiner, 2005; Van Orden et al., 2010).

Together, these findings highlight the significant public health concern of unmet belongingness needs as reflected in elevated loneliness, most recently identified as a public health crisis by the U. S. Surgeon General (2023). Given the amount of time most adults spend working, enhancing workplace social connections through increased social support can promote positive mental health. Furthermore, given the significant impact of the supervisory-employee relationship on well-being, targeting supervisor social support is an important point of intervention (e.g., Arnold, 2017; Hammer et al., 2007; Hancock et al., 2023). This is because the short- and long-term consequences of loneliness are profound and impact not only the employee’s own mental health, but also spills over to co-workers and the larger organization. The workplace also affords valuable opportunities through organizational perceived support (POS; Eisenberger) and specifically through direct supervisors and managers in bolstering social connection and social relationships (Hammer et al., 2022; Hancock et al., 2023). The essential elements for building social connection, i.e., a stable and enriching set of regular and ongoing social interactions, can be found in the workplace through supervisor and co-worker interactions (Reich & Hershcovis, 2011), which can be the key to improving belongingness need satisfaction by building more positive social relationships (Allen & Eby, 2007; Baumeister & Leary, 2017). Positive relationship development hinges on multiple dimensions including support, or the degree to which the relationship is built upon empathy, trust, concern for others, and behavior reflecting loyalty (Ferris et al., 2009). Social support theoretical models (Bavik et al., 2020; Cohen & Wills, 1985) provide a robust framework for understanding the functions and processes of support provision and offer guidance for interventions aiming to improve relationships at work that help meet belongingness needs.

Social support includes emotional, as well as instrumental or more tangible forms of assistance (e.g., Cutrona, 1989; House, 1981), and while low social connectedness is an established risk factor of poor mental health (Joiner et al., 2009), it can be reduced through increased social support training (Mohr et al., 2023). Therefore, we argue that supervisor social support can serve as a *key resource* or buffer against the deleterious effects of workplace stress or strain on employee mental health, and is consistent with social support theory (Cohen & Wills, 1985) through an increase in social connection, belongingness, and providing personal resources to employees.

This role of social support as a resource is consistent with the Job Demands Resources (JD-R) model (Bakker et al., 2007; Demerouti et al., 2001), in which resources buffer the effects of job demands on stress and strain-related outcomes. According to the JD-R model (Demerouti et al., 2001), accumulated resources, which include social resources from relationships, are inversely related to job disengagement. Subsequent work demonstrated that resources also directly influence job demands (Demerouti et al., 2019). According to Bakker and Demerouti, (2007) “resources are not only necessary to deal with job demands, but they also are important in their own right” (p. 312). As Bakker and Demerouti note, this conceptualization of resources is also consistent with the Conservation of Resources (COR) Theory (Hobfoll, 1989; Hobfoll et al., 2018), which articulated an alternative model to stress focusing on people’s underlying drive to build and accumulate resources and monitor resource gains.

Thus, supervisors, managers, and leaders play a significant role in contributing to employee mental health (Arnold, 2017; Hancock et al., 2023; Inceoglu et al., 2018; Kelloway & Barling, 2010), partially due to their discretion to enact policies and distribute resources (Hammer et al., 2023a), and partially due to their constructive or destructive social relationships with employees (Hancock et al., 2023). This is also consistent with a recent systematic review of social support in the management sciences that points to the role of social support as a positive catalyst that can facilitate employee mental health (Bavik et al., 2020). While much of this work has been correlational, a recent review of randomized controlled trials evaluating the effectiveness of supervisor/managerial training interventions aimed at reducing occupational stress and improving well-being of workers further confirmed that supervisors play an important and untapped role in improving mental health by reducing risk factors.

In sum, social support and resource theories suggest that a modifiable mechanism in workplaces to reduce resource loss and increase resource gains associated mental health is to increase supervisor social support. Social support is also a way to combat loneliness and lack of social connection that have been considered major public health concerns (Cacioppo & Cacioppo, 2018; U.S. Surgeon General, 2023), both in the U.S. and internationally (Surkalim et al., 2022). Finally, a recent meta-analysis has demonstrated the importance of supervisor/manager resources impacting employee well-being (Lesener et al., 2019). Thus, we suggest that supervisor support promotes employee mental health and protects against the potential stress and resource loss associated with poor mental health and mental disorders.

## Framework for Protecting and Promoting Employee Mental Health: Mental Health Supportive Supervisor Behaviors (MHSSB)

Given clear evidence on the role of supervisors in employee mental health (e.g., Arnold, 2017; Hancock et al., 2023; Kelloway et al., 2023; Lesener et al., 2019), our framework centers social support from supervisors and managers as an important target for promoting and protecting mental health. Drawing on social support theory (Cohen & Wills, 1985), job strain theory (Karasek, 1979), JD-R (Demerouti et al., 2001) and COR (Hobfoll, 1989) theories, as well as the accumulated evidence on the importance of social connections to psychological health and well-being (e.g., Holt-Lunstad et al., 2021), we describe here the proposed mental health supportive supervisor behavior (MHSSB) framework.

Leaders and supervisors can impact on employees’ mental health in both positive and negative ways (e.g., Arnold, 2017; Hammer et al., 2023a; Hancock et al., 2023; Inceoglu, et al., 2018). Here we focus on the role of the supervisor and manager in promoting mental health of employees because training leaders to increase their social support for employees has been one of the only evidence-based interventions to demonstrate promise in this area (see Kossek et al., 2019; Mohr et al., 2023), as well as demonstrating impact on more general daily well-being (Mohr et al., 2021). Furthermore, recent workplace mental health guidelines from the WHO (2022) suggest the need for managerial training to support employee mental health at work, and the U.S. Surgeon General’s (2023) report on the loneliness crisis suggests that supervisor supportive strategies may be ideal for targeting training through increasing belonging and reducing loneliness at work. Likewise, the recent Office of the U. S. Surgeon General (2022) Workplace Mental Health Framework emphasizes the importance of the supervisor role in promoting mental health at work. These public health documents identify the importance of supervisor supportive strategies, more generally, as central to protect the mental health of our workforce.

Based on specific strategies identified through previous supportive supervisor training interventions (e.g., Hammer et al., 2011; 2021) and mental health awareness training (MHAT) for managers (e.g., Dimoff & Kelloway, 2019a; Dimoff et al., 2016a, b), we suggest that MHSSB are comprised of six strategies that can be easily trained and enacted by supervisors, including three proactive strategies: (1) emotional support, (2) practical or instrumental support, and (3) role modeling, as well as three mental health-specific responsive strategies: (4) stigma reduction, (5) warning sign recognition, and (6) warning sign response (see Table 1).

## Development of MHSSBs

In a line of research on Family Supportive Supervisor Behaviors (FSSB; Hammer et al., 2009), Hammer et al. (2011) demonstrated in a quasi-experimental study of grocery store workers and their supervisors, when supervisors were trained on FSSB strategies including emotional support, instrumental support, role modeling, and creative work-family management, their employees reported improvements in job satisfaction and physical health, and reductions in turnover intentions, especially when work-family conflict was high for those employees at baseline. Additionally, it was also demonstrated by Hammer et al., (2019) that training supervisors on engagement in social support behaviors; specifically, increasing support for military veterans returning to the workplace following deployments, led to improvements in employee (veteran) reports of health, performance, and job satisfaction. The training also decreased turnover intentions, especially among employees who had high levels of support from their supervisors at baseline. Additionally, in a randomized controlled trial (RCT) evaluating supervisor social support training, Kossek et al. (2019) found that the supportive supervisor computer-based training coupled with strategies to increase employee control over work, was most effective at reducing psychological distress for those employees who had caregiving demands (i.e., high needs) compared to those who did not. Finally, Hammer et al. (2021) found that training supervisors on FSSBs and sleep leadership supportive behaviors led to employee reports of improved job satisfaction, reduced turnover intentions and reduced stress before bedtime in an RCT, and these effects were mediated by employee reports of increased supervisor support for sleep health (i.e., sleep leadership).

## Proactive MHSSB Strategies

Supervisor-based approaches that are focused on training supervisors to engage in what we are referring to as proactive support strategies that take a preventative approach include: demonstrating care (i.e., emotional support), ensure employees have access to resources and tools (i.e., practical support), and supervisors role modeling through sharing how they attend to their own self-care and seek support when needed (i.e., role modeling).

These strategies both protect and promote employee mental health and are based on social support and resource-based theories (e. g., COR; JD-R). The behaviors can be taught to supervisors, managers and leaders to help them improve employee sense of social connection and belongingness. s Below we identify *MHSSB* strategies that can be targeted in managerial training to protect and promote employee mental health and can also be used as a basis for assessing the state of employees’ perceptions of mental health support received from supervisors.

### Emotional Support

Emotional support teaches managers the importance of showing that they hear and understand employees’ work and non-work demands (e.g., Hammer et al., 2009). This is generally focused on improving perceptions that one is being cared for and that one’s feelings are being considered. This may include supportive strategies such as engaging with workers to learn more about their non-work lives, expressing concern for the way that work responsibilities affect non-work, and demonstrating respect, understanding, empathy, and sensitivity with regard to non-work responsibilities. For example, a manager can show emotional support very simply by asking employees about their weekend plans on Friday, actively listening to their response, and then following-up on Monday to find how their plans had gone. A more complex example of emotional support might involve kindly inviting an employee to share more information if they mention that they are feeling unfulfilled and overwhelmed with their work tasks during a performance review. Increasing psychological safety (i.e., shared feelings that employees feel safe at work in sharing concerns and taking interpersonal risks; Edmondson, 1999) is an outcome of providing emotional support, as employees become more comfortable in communicating with managers.

### Practical Support

Practical or instrumental support, on the other hand, is related to behavioral types of support in the form of providing general resources and making sure that employer-provided resources related to mental health are communicated. This includes clarifying Employee Assistance Program (EAP) access and use of family and medical leaves. Practical support involves managers making practical adjustments to help employees meet ongoing workplace and personal demands and may entail reducing work pressures and redesigning jobs. It is the extent to which supervisors provide day-to-day resources or services to assist employees in their efforts to successfully manage their dual responsibilities in work and non-work roles. For instance, if an employee reveals that they are feeling overwhelmed and unfulfilled with their work tasks, a supervisor can engage in practical support by brainstorming and making adjustments to employees’ tasks and responsibilities. This type of support can be proactive or reactive, depending on whether it is provided more generally as information on policies and programs to all employees, or if provided responsively following recognition that someone needs to access mental health resources. Furthermore, this support can include suggesting or responding to scheduling requests for flexibility, interpreting policies and practices, and managing routine work schedules to ensure that employees’ job tasks get done.

### Role Modeling

Role modeling involves demonstrating by example that supervisors and managers are taking care of their own mental health and well-being. This relates to the extent to which supervisors engage in behaviors that employees believe will lead to desirable mental health outcomes, such as leaving work at reasonable hours and taking vacations. Social learning theory states that the vast majority of human learning occurs through the observation of others rather than through direct experience (Bandura & Walters, 1977). Thus, if employees observe their supervisors as modeling behaviors that are supportive of their own mental health, and even observing leaders disclosing their own struggles with mental health, they may feel it is more acceptable to do the same. For example, if a supervisor has struggled with high levels of stress and has successfully used the EAP to receive support, the supervisor could share their experience with employees to remind them that resources exist and can be helpful. In doing so, role modeling helps normalize and destigmatize self-care and help-seeking. Furthermore, organizational cultural change will occur only when supervisors reinforce positive mental health supportive behaviors through what they say and do. This type of role modeling by supervisors and managers can also help to reduced stigma and increase the self-efficacy of stigmatized people in organizations (Stone et al., 2023). Reducing stigma is also discussed below in more detail, as a responsive strategy. It is also important that the organizational culture supports leading by example so that supervisors’ supportive mental health behaviors are seen as beneficial and rewarded, by their employees.

### Responsive MHSSB Strategies

In addition to engaging in proactive, preventative support related to employee health and well-being, training programs specific to mental health have demonstrated the importance of the responsive strategies of reducing the stigma surrounding mental health disorders, recognizing warning signs, and responding to warning signs. Some of this training has been developed for the general public, such as Mental Health First Aid (e.g., Hadlaczky et al., 2014), and Question, Persuade, Respond (e.g., QPR; Litteken et al., 2018) gatekeeper training, both of which have been associated with increased mental health literacy and reductions in stigma surrounding mental health disorders, although no evidence exists on their ability to reduce mental health risk factors (Richardson et al., 2023). Workplace training designed to specifically target managers’ knowledge and understanding of mental health has also been associated with reductions in stigma, with some research indicating that reductions in stigma are critical to future behavioral change among supervisors and increased help-seeking among employees (see Dimoff et al., 2016a, b; Milligan-Saville et al., 2017).

### Reducing Stigma

People in workplaces who experience mental health challenges have been stigmatized, similar to people who are stigmatized based on race, gender, and physical disabilities (Stone et al., 2023). While such stigmatization varies based on the degree of mental health disorder, reducing such stigma is critical to afford people with mental health challenges the same workplace opportunities as others. Thus, reducing stigma involves setting the tone and increasing the psychological safety for employees at work, which in turn facilitates help-seek regarding mental health issues when needed. Research shows that employees are more likely to seek help for physical health issues than mental health issues (Britt, 2000), and mental health issues such as anxiety and depression influence perceptions of employees’ suitability for jobs (Stone et al., 2023). For these reasons, as many as 50% of workers say they would not disclose a mental diagnosis to their employer due to the fear of negative repercussions at work (American Psychiatric Association, 2019). Furthermore, certain higher-risk occupations such as airline pilots and flight controllers still have negative repercussions associated with taking pharmacological drugs associated with mental health conditions. Therefore, policies and practices must be readily available and highly destigmatized in order to increase mental health treatment utilization rates (Corrigan, 2018; Dimoff & Kelloway, 2019b). By improving awareness through training organizational leaders (Stone et al., 2023), and providing information on access to mental health resources, while reducing associated stigma, employers can improve the culture of support around mental health, and help-seeking behaviors. For instance, if an employee discloses to their supervisor that part of the reason they may be feeling overwhelmed with work is because they have recently been diagnosed with an anxiety disorder, their supervisor could respond in a non-stigmatizing way by thanking the employee for sharing that information, reminding the employee of workplace resources related to mental health and general well-being, and asking the employee what they (the supervisor) might be able to do to provide support.

Unfortunately, a recent Cochran Report concluded that training programs designed purely to improve mental health knowledge and reduce stigma may be limited in their capability of actually improving mental health outcomes (Richardson et al., 2023). Prior manager training (see Dimoff et al., 2016a, b; Dimoff & Kelloway, 2019a) related to mental health in the workplace demonstrates the importance of going beyond mental health literacy (i.e., knowledge about mental health and mental health disorders) to train managers to recognize and respond to warning signs that an employee is experiencing deteriorating mental health.

### Warning Sign Recognition

Mental health literacy is a critical foundation for any mental health training for the workplace, as managers must not only have a basic understanding of mental health and mental health conditions, but they must also be able to recognize when employees are struggling (Dimoff & Kelloway, 2019a, b). Validated tools, such as the Signs of Struggle (SOS) measure, provide managers with a list of behavioral warning signs that correlate with poor mental health and help managers identify how to detect patterns of behavioral changes that indicate an employee is struggling with high levels of stress or strain and may not be performing optimally (Dimoff & Kelloway, 2019b). For instance, if an employee is usually very social and has shown a pattern of enjoying being very busy on the weekends but then gradually seems less social and more withdrawn, repeatedly sharing that they have no plans, it may be a sign that the employee is struggling with a decline in mental health. The supervisor may also notice that the employee is less engaged with coworkers, contributing less than usual during team discussions, or even keeping their camera off during virtual meetings. When combined with validated training, tools such as the SOS, can help managers initiate supportive conversations where they remind employees about resources and sources of support in the workplace, such as accommodations, EAP, and, if needed, leave (e.g., sick leave, personal leave, disability leave).

### Warning Sign Response

Responding to a warning sign frequently involves actively responding and making accommodations for employees in terms of their work and/or work schedule. While accommodations for many common physical health issues, such as carpal tunnel syndrome and tendinitis, are widely utilized and accepted in many workplaces following a physical health disclosure, workplace accommodations should also be available and accessible for supporting mental health. Some accommodations are similar to those appropriate for physical health issues, such as more frequent breaks, workstation adjustments, and temporary changes to duties and tasks. For instance, if an employee has been socially withdrawing at work or has disclosed that they have been diagnosed with an anxiety disorder, their supervisor should not only respond with empathy, but also with a discussion with the employee about accommodation options or suggestions on other sources of support, such as Human Resources. If accommodations are put into place or if an employee takes leave (e.g., sick leave), supervisors must also understand how to communicate with the employee and provide accommodation when they return-to-work (Dimoff & Kelloway, 2019b).

## Summary of MHSSB

In response to the growing mental health crisis and the corresponding increased interest in workplace mental health by organizational practitioners, we offer this integrated framework for guiding manager and supervisor training related to increasing support for employee mental health at work. Such supportive strategies are both theoretically- and empirically-based and provide policy makers, researchers, and practitioners with clear content that is easy to apply and based on common sense and straightforward tips and behaviors. While numerous toolkits and strategies for navigating workplace mental health at work are available, none appear to be as deep-seated in science as this framework presents.

To our knowledge, only two workplace mental health interventions have been developed that are directly focused on *manager/supervisor* mental health strategies. The first is the online Workplace Mental Health Training (WMHT) for managers that incorporates each MHSSB described in this framework: emotional support, practical support, role modeling, reducing stigma, recognizing warning signs, and responding to warning signs. The WMHT training was originally designed to teach active-duty military leaders to support psychological health, resilience, and overall well-being of their soldiers, was tested in a rigorous RCT, and resulted in positive reactions and improvements in leader’ learning/knowledge by the trainees themselves, as well as reduced anger among their soldiers (Hammer et al., 2023b). Furthermore, Mohr et al. (2023) found that the WMHT led to reduced loneliness four months following the training, and improved employee reports of supervisor emotional support and stigma-reducing behaviors among those with higher baseline loneliness. The training was also found to be most effective for those soldiers experiencing higher levels of stress at baseline based on their reports of increased supervisor practical support, role modeling support, stigma reduction, warning sign recognition, and improved supervisor response to warning signs at follow-up, compared to those soldiers with lower levels of stress at baseline (Dimoff et al., 2023). This training is currently being customized for a non-military workforce and is being pilot tested, demonstrating promising results.

The second is the Managing Minds at Work online training program (Blake et al., 2022; Thomson et al., 2023). This program is also online and incorporates most of the MHSSBs, while primarily focusing on improving managers’ own mental health, understanding ways of redesigning work to promote employee mental health, improving psychological safety for employees and the associated conversations needed. Effectiveness data thus far demonstrates positive impacts on manager behavioral intentions related to preventing declines in mental health and protecting positive mental health, however mental health-related data from employees has not yet been examined (Blake et al., 2022; Thomson et al., 2023).

## Future Research and Practice Needs

We have identified three areas for future research and practice on workplace mental health based on the MHSSB framework. We first discuss supervisor workplace mental health training needs. Second, we review the role of co-workers in supporting employee mental health. This is followed by important implementation science issues that are necessary to consider going forward if we want to truly advance the field of workplace mental health.

## Supervisor Workplace Mental Health Training Needs

Evidence-based interventions to reduce or prevent adverse mental health outcomes at work are almost non-existent, and of those that do exist, most are focused on individual-level/tertiary prevention, as opposed to organizational-level/primary prevention strategies (e.g., Anger et al., 2024; Aust et al., 2023; Edgelow et al., 2022; Rosado-Solomon, 2023). Even fewer trainings have focused on building manager skills to support employees and only two approaches, to our knowledge, integrate proactive and responsive supportive strategies in such trainings (Hammer et al., 2023b; Thomson et al., 2023). One of these trials even provides effectiveness data demonstrating significant positive impacts on manager learning and reaction criteria, as well as on employee reductions in anger and loneliness, important risk factors for mental health challenges (Hammer et al., 2023b; Mohr et al., 2023).

Additionally, the WHO has recognized the importance of training managers to support workers’ mental health in their evidence-based workplace mental health guidelines released in 2022, which also emphasizes the need for mental health literacy and awareness for all workers. Despite research evidence that points to the important role of managers in impacting employee mental health and well-being (Arnold, 2017; Hammer et al., 2007; Hancock et al., 2023; Inceoglu et al., 2018; Kelloway & Barling, 2010; Mohr et al., 2021, 2023), we conclude there is little systematic research on the effectiveness of such manager training approaches. Thus, calls for supervisor training on workplace mental health (WHO, 2022), and specific suggestions for research on the effects of such training on reducing mental health stigma in workplaces (Stone et al., 2023), affirm the need for training managers and supervisors on how to support employee mental health, and ideally utilizing the MHSSB framework.

Furthermore, we suggest that future research compares the effectiveness of the various proactive and responsive forms of support on mental health. This could include comparing the effectiveness of supervisor emotional support to effectiveness of stigma reduction on employee mental health outcomes. Future research should also investigate ways of increasing the strength of the training, for example, by evaluating the effectiveness of different training platforms such as computer-based training, webinars, and face-to-face training in determining effectiveness outcomes. Thus, we suggest there is a need for manager training interventions that integrate these MHSSBs for improving mental health of employees to help advance research, policy, and practice.

## The Role of Co-Workers in Workplace Mental Health

In addition to manager support training, co-worker/peer support is an important protective factor for employee mental health (Edgelow et al., 2022), and an area of needed research. Support from peers provides a foundation for social connection through shared lived encounters and validations of ones’ experiences (Gillard et al., 2017). Peer support workers have a positive and profound influence on individual mental health (Repper & Carter, 2011), and therefore, co-worker/peer support offers a strategy to assist employees with resources and skills to reduce stigma and stress and improve mental health at work in order to promote healthier, happier and more productive lives within and outside of work. Unfortunately, little information exists on effective mental health co-worker supportive strategies.

Correlational evidence suggests that co-worker support is related to fewer depressive symptoms (Perry-Jenkins et al., 2011), lower psychological distress (López Gómez et al., 2019), and day-level mental health (Simbula, 2010). Yet, few systematic studies exist on identification and evaluation of effective co-worker support strategies, interventions, or training. One exception is the Coworker Health Awareness Training (CHAT), a two-hour program designed to help employees become more knowledgeable about mental health and better support their coworkers in a workplace environment (Oakie et al., 2018).

What does exist has limited effectiveness data and primarily represents individual-level intervention strategies (e.g., Paterson et al., 2021). An example is provided by Marks and colleagues (2017) who identified co-workers/peers as being a critical to preventing further decrements in mental health, however have not been shown to promote mental health. Specifically, the authors suggest the importance of training peer-support team members to REACT (Recognize, Evaluate, Advocate, Coordinate, and Track) to observations of mental health strain among co-workers as a strategy to promote positive psychological health. This approach is similar to Mental Health First Aid and QPR in its focus on responsive strategies (e.g., increasing mental health literacy and corresponding ways of recognizing and responding to mental health challenges in co-workers. Evaluation of the REACT peer support training showed significant knowledge increases. In addition to assessing immediate knowledge gains post-training for REACT, Horan and colleagues (2021) assessed first responder intent for peer support conversations and self-efficacy post-implementation of the peer support REACT training, demonstrating a significant increase in both intent and self-efficacy to use supportive peer conversations post-training (Horan et al., 2021). Additionally, the authors found that evidence of pre-training trust in peers and self-efficacy influenced employee self-efficacy for use of the training concepts, but there appears little evidence of mental health promotion and protection based on this model through impacts on mental health outcomes directly.

We suggest that future research is needed on co-worker/peer support strategies to facilitate support at work for improving the well-being of organizations and the workforce as a whole (Jones et al., 2022; Oakman et al., 2020). While current co-worker/peer support for mental health strategies shows positive improvements in knowledge gains and attitudes post-training, future research should assess the impact of co-worker support strategies on additional employee mental health outcomes such as psychological strain, willingness to seek mental health treatment, anger, loneliness, alcohol consumption, and suicidality, for example. Thus, we suggest that more research is needed in this critical area to expand our understanding of the effectiveness of workplace strategies for protecting population mental health through the training of co-workers on social support strategies that extend to proactive/preventative strategies.

## Implementation Science and Workplace Mental Health

Another issue to consider in future research and practice is the importance of implementation science that identifies the barriers and facilitators of mental health interventions in workplaces (e.g., Guerin et al., 2022; LaMontagne et al., 2021; Nielsen & Shepherd, 2022; Yarker et al., 2022). For example, Nielsen and Yarker (2023) noted that process measures specific to the evaluation of mental health interventions should include assessments at different levels of an organization to identify structural and systemic barriers and facilitators to implementation. In their Integrated Training Transfer and Effectiveness Model (ITTEM) Nielsen and Shepherd (2022) integrate the training transfer and training effectiveness literatures suggesting that training context affects both the transfer of training as well as its effectiveness. This model proposes five phases that enables evaluation and transfer to be assessed together: Phase 1, is evaluation of the pre-training context; Phase 2, training content and delivery methods; Phase 3 occurs immediately post-training, where skills and knowledge are translated into actual changes in emotions, cognitions, and behaviors; Phase 4 occurs when training is transfer into *sustained* changes in emotions, cognitions, and behaviors; finally Phase 5 is the sustainability of the trained concepts over time leading improvements in mental health and well-being.

Given the need for evidence-based research to be translated into practice, we also suggest future implementation research be informed by RE-AIM (Reach, Effectiveness, Adoption, Implementation, and Maintenance) (Glasgow et al., 1999), providing a comprehensive conceptual framework for identifying and understanding effectiveness, perceived feasibility and barriers to implementation. We suggest that there is a clear need to research and understand organizational barriers to implementation and adoption that limit sustainability of such interventions. Interestingly, occupational health psychology researchers have rarely partnered with implementation scientists to conduct pragmatic trials that establish the effectiveness of interventions along with the collection of data to identify how best to implement these programs in real-world organizations (Dugan & Punnett, 2017; Guerin et al., 2022).

## Summary

We suggest that the workplace is athe *missing link* in public health mental health protection (Hammer et al., 2022). We provide a framework that is evidence-based and offers six strategies for workplace mental health supervisor training, MHSSBs (i.e., emotional support, practical support, role modeling, reducing stigma, warning sign recognition, warning sign response), known to protect and promote employee mental health. This is accomplished through providing supervisors and managers training that increasesesources to increase their social support and connectedness with employees, and in turn, decreases reports of employee loneliness and help-seeking stigma, and improves their perceived social support and sense of belongingness. Given that work is a social determinant of health (e.g., Lovejoy et al., 2021; Niedhammer et al., 2021), we suggest that training supervisor supportive strategies that target reductions in workplace psychosocial risk factors, and increases in protective factors, with the goal to promote positive mental health at work, will thereby contribute to preventing chronic mental disorders in our population.

Despite the history of organizational science research on worker chronic health conditions (e.g., McGonagle & Barnes-Farrell, 2014) and mental health specifically (e.g., Chari et al., 2018), employers have only recently begun investing ways the workplace can promote employee mental health, support workers with chronic mental health conditions, and reduce poor mental health outcomes (Kelloway et al., 2023). We believe that it is imperative that the impact of the workplace on mental health be better understood and prioritized by employers. Managers and co-workers play a vital role in workplace occupational health and safety (Nielsen & Randall, 2009), and their role may be even more critical when it comes to mental health. As workplace authorities, managers can help destigmatize use of resources and accommodations, increase awareness of sources of support, and provide practical support to employees by acting as gatekeepers who direct employees to resources and benefits (Kelloway et al., 2023; Nielsen & Randall, 2009). The extent to which employees are willing to seek out resources can be highly dependent on their managers’ supportive (or unsupportive) behaviors (e.g., Hancock et al., 2023), and the psychological safety employees feel. As suggested by the WHO, manager training is central to ensuring that employee mental health is supported and that broader mental health policies and programs are implemented, endorsed, and used by employees (WHO, 2022).

Workplaces need to move to multi-level approaches of supporting mental health at work by engaging top management, front-line managers and supervisors, and employees, in understanding and implementing prevention strategies to improve workplace mental health and reduce chronic mental disorders in our population.

## Figures and Tables

**Table 1:** Overview of workplace Mental Health Supervisor Supportive Behaviors (MHSSB)

Support behavior	Definition	Example behaviors	Conversation starter

Emotional Support	Support that shows you care about your employees’ mental health	• Increase connection between you and your employees by increasing face-to-face contact • Convey genuine concern about the mental health of your employees	How are you doing today?
Practical Support	Making practical arrangements to meet the needs of employee’s personal demands	• Discuss the mental health resources available to employees during team meetings • Make arrangements and adjust employee schedules to reduce conflict with personal responsibilities and challenges	As your supervisor, how can I adjust your schedule to best suit your personal needs and challenges?
Role Modeling	Lead through example by modeling how you take care of your own mental health and well-being	• Share healthy coping strategies that have helped your mental health • Be clear and prioritize your mental health needs as a supervisor	This is how I manage my mental health and what has worked for me, what might work for you?
Stigma Reduction	Clearly communicating that it is healthy, safe and normal to seek help and support regularly and when in need	• Normalize seeking help and support and emphasize workplace resources • Provide a safe environment by being discrete about employee well-being conversations and challenges	I want to let all of my team members know about the following workplace resources to support your mental health. I have had great experience myself with these resources.
Warning Sign Recognition	Recognizing when an employee may be struggling with their mental health	• As a supervisor you recognize when your employee is upset about something • You recognize when an employee is not themselves	I noticed that you missed our team meeting this morning, is everything okay?
Warning Sign Response	Actively responding when you observe an employee who may be struggling with their mental health and well-being	• Regularly remind your employees that you are there to help • Use open-ended questions when asking employees about their mental health	Is there a time that works best for you to check-in today?
